# Loss of Axon Bifurcation in Mesencephalic Trigeminal Neurons Impairs the Maximal Biting Force in Npr2-Deficient Mice

**DOI:** 10.3389/fncel.2018.00153

**Published:** 2018-06-15

**Authors:** Gohar Ter-Avetisyan, Alexandre Dumoulin, Anthony Herrel, Hannes Schmidt, Johanna Strump, Shoaib Afzal, Fritz G. Rathjen

**Affiliations:** ^1^Max-Delbrück-Center, Berlin, Germany; ^2^Département Adaptations du Vivant, UMR 7179 Centre National de la Recherche Scientifique/MNHN, Paris, France

**Keywords:** axonal branching, mesencephalic trigeminal neurons, cGMP signaling, Npr2, CNP

## Abstract

Bifurcation of axons from dorsal root ganglion (DRG) and cranial sensory ganglion (CSG) neurons is mediated by a cGMP-dependent signaling pathway composed of the ligand C-type natriuretic peptide (CNP), the receptor guanylyl cyclase Npr2 and the cGMP-dependent protein kinase I (cGKI). Here, we demonstrate that mesencephalic trigeminal neurons (MTN) which are the only somatosensory neurons whose cell bodies are located within the CNS co-express Npr2 and cGKI. Afferents of MTNs form Y-shaped branches in rhombomere 2 where the ligand CNP is expressed. Analyzing mouse mutants deficient for CNP or Npr2 we found that in the absence of CNP-induced cGMP signaling MTN afferents no longer bifurcate and instead extend either into the trigeminal root or caudally in the hindbrain. Since MTNs provide sensory information from jaw closing muscles and periodontal ligaments we measured the bite force of conditional mouse mutants of Npr2 (*Npr2*^*flox*/*flox*^*;Engr1*^*Cre*^) that lack bifurcation of MTN whereas the bifurcation of trigeminal afferents is normal. Our study revealed that the maximal biting force of both sexes is reduced in *Npr2*^*flox*/*flox*^*;Engr1*^*Cre*^ mice as compared to their *Npr2*^*flox*/*flox*^ littermate controls. In conclusion sensory feedback mechanisms from jaw closing muscles or periodontal ligaments might be impaired in the absence of MTN axon bifurcation.

## Introduction

Axonal branching is crucial for normal brain function, and impairments of branching might result in neurological or neurodevelopmental disorders (Bullmore and Sporns, 2009; Nugent et al., 2012; Chédotal, 2014). Although axon branching has been the subject of intense studies, our knowledge of the underlying molecular mechanisms is still fragmentary (Schmidt and Rathjen, 2010; Gibson and Ma, 2011; Kalil and Dent, 2014; Winkle et al., 2016; Armijo-Weingart and Gallo, 2017; Dumoulin et al., 2018). Analysis of the branching of sensory axons in the spinal cord and hindbrain unraveled a cGMP signaling cascade to be essential for bifurcation—a specific form of axon branching characterized by the splitting of the growth cone. The cGMP signaling cascade is composed of the ligand CNP (C-type natriuretic peptide), the receptor guanylyl cyclase Npr2 (natriuretic peptide receptor 2, also designated guanylate cyclase-B) and the cGMP-dependent kinase I (cGKI, also termed PKGI) (Schmidt et al., 2002, 2007, 2009; Zhao and Ma, 2009; Zhao et al., 2009; Xia et al., 2013; Ter-Avetisyan et al., 2014). In the absence of CNP, Npr2 or cGKI in mouse models dorsal root ganglion (DRG) or cranial sensory ganglion (CSG) axons do not bifurcate when entering the spinal cord or the hindbrain, respectively. Instead, they either turn in an ascending or descending direction. Collateral formation from these stem axons is not impaired (Schmidt et al., 2007, 2009; Ter-Avetisyan et al., 2014) and might be regulated by different mechanisms (Tymanskyj et al., 2017). Variations in cGMP levels caused by the absence of phosphodiesterase 2A—the main species of cGMP hydrolyzing enzymes in embryonic DRG neurons—do not interfere with proper bifurcation of sensory axons (Schmidt et al., 2016). Loss of axon bifurcation due to the conditional inactivation of *Npr2* in DRG neurons leads to altered termination fields of primary afferents from the skin in the spinal cord (Tröster et al., 2018). Furthermore, behavioral testing of conditional *Npr2* mouse mutants gave evidence that noxious heat perception and nociception induced by chemical irritants are impaired in the absence of axon bifurcation of DRG neurons, whereas responses to mechanical stimulation and motor coordination are surprisingly normal (Tröster et al., 2018).

Here we aimed to identify projecting axons that use the CNP/Npr2/cGMP/cGKI signaling cascade to bifurcate in specific regions of the developing mouse brain. Therefore, we analyzed the CNP, Npr2, and cGKI expression profiles using reporter mice for Npr2 and CNP and antibodies against cGKI at early developmental stages when scaffolds of axon tracts are generated in the brain (Easter et al., 1993; Mastick and Easter, 1996; Molle et al., 2004). We demonstrate that mesencephalic trigeminal neurons (MTNs, also abbreviated as MesV or Me5) of the midbrain express Npr2 and cGKI. MTNs are the first-born neurons of the midbrain and like DRG neurons, they are pseudo-unipolar. MTN afferents initially project ventrally and then turn at 90 degrees to extend caudally. In the mouse their descending afferents pioneer the lateral longitudinal fasciculus during embryonic development followed by axons of the medial longitudinal fascicle (Chédotal et al., 1995; Mastick and Easter, 1996; Molle et al., 2004; Ware and Schubert, 2011). The MTN afferents cross the isthmus to extend within the basal plate of the hindbrain and bifurcate at the level of the trigeminus (Shigenaga et al., 1988; Luo et al., 1991). One branch exits the hindbrain, passes through the trigeminal ganglion (gV) (Stainier and Gilbert, 1990) and extends to the jaw. The other branch stays within the hindbrain and projects to the trigeminal motor nucleus (abbreviated Vmo or Mo5) and to the supratrigeminal nucleus (Vsup) (Widmer et al., 1998; Yoshida et al., 2017). In mammals 80–90% of MTNs innervate spindles of jaw closing muscles (masseter, temporalis) and 10–20% form mechanoreceptors within the periodontal ligaments (Turman, 2007). MTNs process proprioceptive information from these structures and thus play an essential role in coordinating biting, ingestion and mastication (Dessem and Taylor, 1989; Hunter et al., 2001; Lazarov, 2007).

By axon tracing using DiI or a genetic sparse labeling method to trace the path of MTN afferents we demonstrate that in the absence of the ligand CNP or the receptor Npr2, MTN axons do not bifurcate in rhombomere 2 of the hindbrain in global and conditional mouse knockouts. Since MTNs are implicated in oral-motor activities we tested the functional consequences of the loss of bifurcation by measuring the maximal bite force of mice in which Npr2 was conditionally inactivated by *Engrailed1-Cre* (*Engr1-Cre*). We found a reduction in the maximal bite force in mutant mice which might be explained by impaired sensory feedback mechanisms from masseter muscles or periodontal ligaments to trigeminal motor neurons of the hindbrain.

## Materials and methods

### Mice

Heterozygous *Npr2-LacZ* [B6.129P2-*Npr2*^*tm1.1*(*nlsLacZ*)^/Fgr] (Ter-Avetisyan et al., 2014) and heterozygous *CNP-LacZ* [B6;129P2-*Nppc*^*tm1.1*(*nlsLacZ*)^/Fgr] (Schmidt et al., 2009) mice were used to monitor the expression of Npr2 and CNP, respectively, in embryonic brains. Both transgenic lines encode an nuclear localization signal for β-galactosidase. Expression of Npr2 was also monitored in heterozygous crossbreedings of *Npr2-CreERT2* [B6.129S7-*Npr2*^*tm1*(*CreERT*2)^/Fgr] mice (Ter-Avetisyan et al., 2014) and the reporter line *R26-LSL-tdTomato* [B6.Cg-*Gt(ROSA)26sor*^*tm14*(*CAG*−*tdTomato*)*Hze*^/J] which expresses a red fluorescent protein variant (tdTomato) (Madisen et al., 2010) following tamoxifen-induced Cre-recombination of its *loxP*-flanked STOP cassette as previously described (Ter-Avetisyan et al., 2014). To get an overview on the localization of Engr1-Cre in embryonic brains crosses of the mouse line encoding *Engr1-Cre* [B6;129SV-*En1*^*tm2*(*cre*)*Wrst*^/Fgr] (Kimmel et al., 2000) with the reporter line *R26-LacZ* [B6N.129S4(B6)-*Gt(Rosa)26Sor*^*tm1Sor*^/CjDswJ] (Soriano, 1999) or with *R26-LSL-tdTomato* were analyzed.

DiI axon tracing was done in homozygous and heterozygous *Npr2-LacZ* or *CNP-LacZ* mice and in conditionally inactivated Npr2 mice using *Npr2-flox* mouse mutants [B6;129S7-*Npr2*^tm4(*flox*)^/Fgr] (Tröster et al., 2018) crossed with *Wnt1-Cre* [*Tg(Wnt1-cre)*^*11RthTg*(*Wnt*1−*GAL*4)*11Rth*^/Fgr] (Danielian et al., 1998) or with *Engr1-Cre*.

Transgenic sparse labeling of Npr2-positive axons was obtained by crossbreeding *Npr2-CreERT2* mice with the reporter line *Z/AP* [*Tg (CAG-Bgeo/ALPP)1Lbe*] (Lobe et al., 1999) and the spontaneous loss-of-function *Npr2-cn* mouse line (Tsuji and Kunieda, 2005) as described (Schmidt et al., 2013). The latter *Npr2* null allele was crossbred to avoid homozygosity of the *Npr2-CreERT2* allele in homozygous *Npr2* mutants. Genotyping of these mouse strains has been detailed in the above mentioned references. The heterozygosity of the Cre-recombinase or reporter alleles was maintained in mouse breedings.

Animals were housed on a 12/12 h light/dark cycle with free access to food. The animal procedures were performed according to the guidelines from directive 2010/63/EU of the European Parliament on the protection of animals used for scientific purposes. All experiments were approved by the local authorities of Berlin (LaGeSO) (numbers T0313/97, G0370/13, X9014/15 and G0090/16). Littermate mice (15 weeks old) were used in bite force measurements. Animals were habituated to the experimental room and were investigated by observers blinded for the genotype.

### Axon tracing, cell counting and immunhistology

For DiI axon tracing embryos were fixed for 4 h at 4°C in 4% paraformaldehyde/PBS solution at embryonic day 12.5, then transferred in PBS followed by embedding in low melting agarose (3.5%) to cut 200 μm thick slices using a vibratome (Leica VT 1000S). DiI (5% w/v in ethanol; Sigma) was applied with fine-tipped glass pipette under a dissecting microscope in axon fascicles formed by the MTN axons in the rostral region of the hindbrain (about 400–600 μm rostral of the trigeminus entry zone). MTN axon fascicles were identified on the basis of their location in the hindbrain slice. Labelled slices were incubated at room temperature in PBS for a minimum of 5 and up to 10 days. The diffusion of the dye was examined in daily intervals. The analysis at the single axon level was conducted in confocal z-stacks at the level of rhombomere 2 using a Carl Zeiss LSM 710 NLO Laser Scanning Microscope equipped with ZEN 2010 software. MTN axons were identified on the basis of their lateral localization in the hindbrain (see also Figures **3A_2_,_3_**).

A genetic approach for sparse labeling of Npr2-positive axons involving a tamoxifen-inducible variant of Cre-recombinase under control of the *Npr2* promoter (*Npr2-CreERT2*) and the conditional *Z/AP* reporter mouse line has been described (Lobe et al., 1999; Schmidt et al., 2013; Ter-Avetisyan et al., 2014). Reporter gene expression in a subset of Npr2-expressing cells in mouse embryos was obtained by crossbreedings of heterozygous *Npr2-CreERT2* mouse mutants with the reporter line *Z/AP* and subsequent application of 0.1 mg per g body weight tamoxifen by oral gavaging to timed pregnant females at E9.5 as previously described (Ter-Avetisyan et al., 2014). Mouse embryos heterogzygous for *Npr2-CreERT2* and *Z/AP* served as controls. Homozygous mouse mutants for *Npr2* were obtained by additional crossbreeding with mice carrying the *Npr2-cn* null allele (Tsuji and Kunieda, 2005). Embryos were collected at E12.5, fixed in 0.2% glutaraldehyde, 2% paraformaldehyde, 100 mM MgCl_2_ in PBS, transferred to PBS followed by inactivation of endogenous alkaline phosphatase and further overnight incubation in PBS with 100 mM MgCl_2_. Tissue samples were embedded in low melting agarose as described above. Two hundred fifty micrometer thick vibratome sections were stained as detailed elsewhere (Schmidt et al., 2013; Ter-Avetisyan et al., 2014). Due to strong background of the alkaline phosphatase staining in hindbrain vibratome sections and weak expression of Z/AP gene product in MTN axons camera lucida images were prepared in some cases. Afferents of the trigeminal afferents were traced by DiI.

Whole mounts of embryos or cryostat sections (16-μm thick) fixed either in Zamboni's fixative or in 2% paraformaldehyde were stained with X-gal or with the following antibodies as detailed elsewhere (Ter-Avetisyan et al., 2014): a rabbit polyclonal antibody to full length cGKI expressed in eukaryotic cells (0.75 μg/ml) (Ter-Avetisyan et al., 2014), mAb anti-neurofilament (2H3, Developmental Hybridoma Bank), rabbit anti-red fluorescent protein (1:2500, ABIN129578; https://scicrunch.org/resolver/RRID:AB_10781500RRID:AB_10781500), chicken-anti β-galactosidase (Abcam, ab 9361, 1:5,000), rabbit anti-peripherin (Millipore, AB1530, 1:200), mAb Tuj 1 (Sigma T8535, 1:1,000), mAb anti-Islet1/2 (clone 39.4D5, Developmental Hybridoma Bank, 1:150), and mAb anti-Brn3a (Millipore MAB1585, 1:200).

For Western blotting the following antibodies were used: Guinea pig antiserum to the extracellular domain of Npr2 (1:5,000) (Ter-Avetisyan et al., 2014), rabbit antibody to full length cGKIα (1 μg/ml of the IgG fraction), chicken anti-β-galactosidase (1:5,000; Abcam, ab 9361; https://scicrunch.org/resolver/RRID: AB_307210RRID: AB_307210), mouse anti-clathrin heavy chain (0.05 μg/ml; BD Biosciences, 610499; https://scicrunch.org/resolver/RRID:AB_397865RRID: AB_397865), mouse anti-GAPDH (1:7,500; Novus Biologicals, NB300-221; https://scicrunch.org/resolver/RRID:AB_10077627RRID: AB_10077627) and rabbit anti-histone H3 (1:5,000; Abcam, ab 1791). To obtain subcellular fractions of forebrain, midbrain or midbrain/hindbrain from embryonic wildtype (E11.5) or *CNP-LacZ* (E13.5) mice, tissues were homogenized in 0.34 M sucrose supplemented with protease blockers [aprotinin (20 U/μl), leupeptin (5 mM), pepstatin (5 mM), PMSF (1 mM)]. Nuclei were pelleted at 200 × g for 10 min and the resulting supernatant was centrifuged at 100,000 × g for 10 min to obtain a crude membrane pellet and the cytoplasmic fraction in the supernatant. The membrane fraction was solubilized in 1% CHAPS in PBS supplemented with protease blockers. Un-solubilized material was removed by centrifugation at 100,000 × g for 10 min.

Secondary antibodies were applied at the following dilutions: goat-anti-rabbit-Cy3, goat-anti-rabbit-Alexa647, goat-anti-mouse-Alexa647, donkey anti-guinea pig-Cy3, donkey anti-chick-IgY-Cy3, goat-anti-rabbit-Alexa488, goat anti-guinea pig-Alexa488 (all 1:1,000; Dianova), donkey anti-guinea pig-HRP, goat anti-rabbit-HRP and goat anti-mouse-HRP (all 1:20,000; Dianova).

DAPI-positive cells were manually counted in microscopic view fields of 319.8 × 319.8 μm of confocal images taken from 16 μm thick cryostat sections of midbrain or hindbrain from heterozygous *Npr2-LacZ* reporter mice. Sections were stained with nuclear stain DAPI, antibodies to β-galactosidase (to monitor Npr2 expression), cGKI, Brn3a or Islet1. Thirty-two and twenty-six view fields of midbrain and rhombencephalon, respectively, were inspected from three embryos each.

Microscopic images were obtained at room temperature by confocal imaging using a Carl Zeiss LSM 710 NLO Laser Scanning Microscope equipped with ZEN 2010 software and the following lenses: a Plan-Neofluar 10x/0.30 NA objective, a Plan-Achromat 40x/1.40 NA oil objective or a Plan-Achromat 63x/1.40 NA oil objective (all from Carl Zeiss MicroImaging, GmbH). Images were imported into Photoshop CS5 (Adobe) for uniform adjustment of contrast and brightness. Confocal z-stacks were assembled, labeled and converted into videos using Image J/-Fiji. Figures were assembled using Illustrator CS5 (Adobe).

### Bite force recordings

We measured bite force from all individuals using a piezoelectric force transducer (Kistler, type 9203, range ± 500 N Kistler, Winterthur, Switzerland) attached to a handheld charge amplifier (Kistler, type 5995). The transducer was mounted between two bite plates, as previously described by (Herrel et al., 1999; Aguirre et al., 2002; Thomas et al., 2015). The tips of both upper and lower bite plates were covered with a layer of cloth medical tape to provide a non-skid surface and to protect the teeth of the animals. The distance between the bite plates was adjusted to assure the same gape angle (30°) for each individual. Bite force was measured during bilateral incisor biting. Two recording sessions were conducted for each animal, during which at least four bites were recorded. Only the single maximal bite force value was retained for further analysis. Bite forces were measured from littermates of adult males and females of each of the following genotypes: *Npr2*^*flox*/*flox*^*;Engr1*^*Cre*^ and *Npr2*^*flox*/*flox*^. The latter served as controls.

### Statistical analyses of bite measurements

Sample size for biting experiments and for analysis of axon branching was deduced from previously published studies. No further statistical methods were used to predetermine sample size. Sample size is given in the figures or legends. Experiments were done blind with respect to genotype. *Npr2*^*flox*/*flox*^ littermates served as controls.

All data were Log_10_-transformed to render them normal and homoscedastic as required for parametric analyses. Analyses were run in SPSS V25. We first tested whether groups differed in body mass using a univariate ANOVA with body mass as dependent variable, and sex and genotype as fixed factors. To test whether bite force in *Npr2*^*flox*/*flox*^*;Engr1*^*Cre*^ mice differed from that in *Npr2*^*flox*/*flox*^ mice we ran an ANOVA with maximal bite force as dependent variable, and sex and genotype as fixed factors. In Figures **5B,K** raw data are shown.

## Results

### The receptor guanylyl cyclase Npr2 and the serine/threonine kinase cGKI co-localize in embryonic MTN

**Figure 1:**
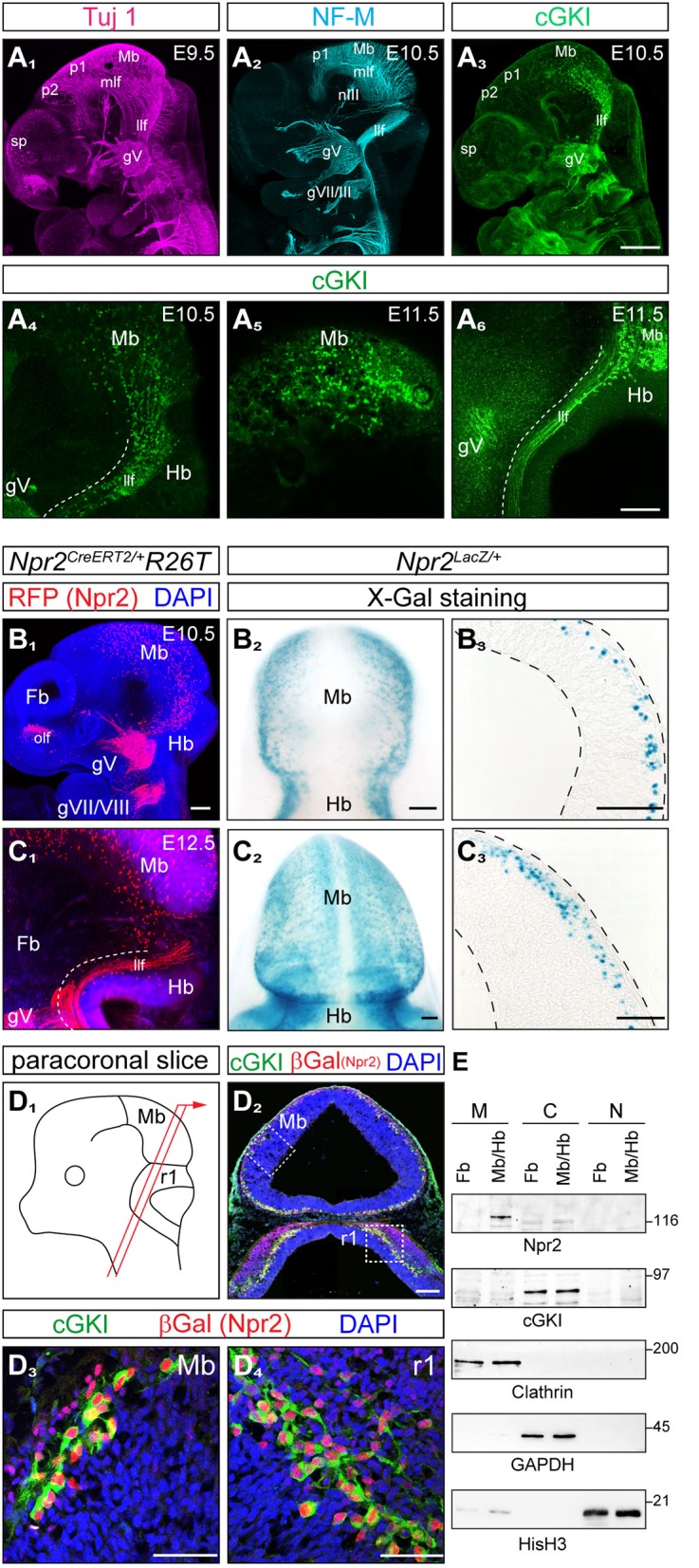
Co-expression of cGKI and Npr2 in the dorsal midbrain and rhombomere 1 at early embryonic stages. **(A)** Analysis of the pattern of expression of cGKI in cells and axon tracts of the mesencephalon at E9.5, E10.5, and E11.5. For comparison, axons tracts labeled by Tuj1 **(A**_1_**)** or neurofilament **(A**_2_**)** from confocal z-stacks are shown. **(A**_3_**-A**_6_**)** Anti-cGKI staining shows the localization of cGKI positive cell somata in the midbrain and descending axons. Scale bar in **(A**_1_**-A**_3_**)**, 200 μm; in **(A**_4_**-A**_6_**)**, 50 μm. **(B)** Detection of Npr2-expressing cells using the *Npr2-CreERT2*-line in combination with the *R26T* reporter line (*R26-LSL-tdTomato*) [in red, counterstained with an antibody to red fluorescent protein (RFP)] or using *Npr2-LacZ* mice by X-Gal staining. Lateral view of a head of a whole mount (**B**_1_; z-stacks), dorsal view **(B**_2_**)** and a sagittal section **(B**_3_**)** of the midbrain are shown at E10.5. Scale bar for **(B**_1_–**B**_3_**)**, 100 μm. **(C)** Expression of Npr2 monitored using the reporter line *R26T* (activated by *Npr2-CreERT2*) or by using the reporter *Npr2-LacZ*. Lateral view of a head in a whole mount preparation (**C**_1_; z-stacks), dorsal view **(C**_2_**)** and sagittal section of the midbrain are shown at E12.5. Scale bar for **(C**_1_–**C**_3_**)**, 100 μm. **(D)** Co-localization of Npr2 and cGKI in a neural population in the dorsal midbrain and rhombomere 1 of cryostat sections at E12.5. Localization of Npr2 was demonstrated by anti-β-galactosidase staining of the *Npr2-LacZ* reporter mouse line. **(D**_3_**)** (mesencephalon) and **(D**_4_**)** (rhombomere 1) show higher magnifications of squares indicated in **(D**_2_**)**. The scheme illustrates the orientation of sectioning **(D**_1_**)**. gV, trigeminal ganglion; gVII, geniculate ganglion; gVIII, ganglion of the vestibulocochlear nerve; Hb, hindbrain; llf, lateral longitudinal fascicle; Mb, midbrain; mlf, medial longitudinal fascicle (rostral to the prosomere 1-mesencephalic boundary); nIII, oculomotor nerve; ofl, olfactory placode; p1, prosomere 1; p2, prosomere 2; r1, rhombomere 1; sp, secondary prosencephalon. Scale bar in **(D**_2_–**D**_4_) 100 μm. **(E)** Western blotting of subcellular fractions of the forebrain and midbrain/hindbrain extracts (E11.5) using antibodies to Npr2 or cGKI. Loading control was obtained by antibodies to clathrin heavy chain (crude membrane fraction), GAPDH (cytoplasmic fraction) or histone H3 (nuclear fraction). M, crude membrane fraction; C, cytoplasmic fraction; N, nuclear fraction. Molecular mass markers are indicated at the right of the panels in kD.

Previous studies revealed co-expression of the receptor guanylyl cyclase Npr2 and the kinase cGKI in peripheral embryonic sensory neurons where both cGMP signaling components are essential for axon bifurcation—a specific form of neuronal branching—in the spinal cord and hindbrain. To identify neuronal populations in the brain co-expressing Npr2 and cGKI we focused on early embryonic stages when major axon tracts are formed. Axons from such Npr2- and cGKI-positive neurons might use CNP-induced cGMP signaling to bifurcate in specific regions of the brain. For comparison Tuj 1 or neurofilament stainings were done to label the scaffold of axon tracts that forms at early embryonic stages (Figures 1A_1_,_2_). Expression of cGKI was analyzed by anti-cGKI antibodies. Whole mount immunostainings of E10.5 and E11.5 mouse embryos detected cGKI positive neurons in the dorso-caudal mesencephalon (Figures 1A_3_,_5_,_6_) and axons descending into the hindbrain (Figure 1A_3_,_4_,_6_). These axons form the lateral longitudinal fasciculus (compare anti-cGKI staining with that of antibody Tuj1 or neurofilament, Figures 1A_1_,_2_). Additional cGKI-positive structures were observed at the border of the secondary prosencephalon. Cells or axons in the rostral part of the mesencephalon, in the developing diencephalon including neurons that contribute to the medial longitudinal tract, the forebrain with neurons of the developing postoptic commissure and the oculomotor nerve were negative for cGKI at these early embryonic stages (Figures 1A_1_-A_6_). In contrast to this restricted pattern of localization cGKI is more widely expressed at postnatal and mature stages suggesting multiple roles in cGMP signaling (Feil et al., 2005).

The localization of the receptor Npr2 in embryonic brains was analyzed by two distinct reporter mouse lines: (1) by a CreERT2-activated reporter *R26-LSL-tdTomato* (*Npr2*^*CreERT2*/+^*;R26T*) (Figures 1B_1_,C_1_) in which the reporter is found throughout Npr2-positive cells including their axons and (2) by the Npr2-LacZ reporter mouse (*Npr2*^*LacZ*/+^) (Figures 1B_2_,_3_,C_2_,_3_) in which β-galactosidase is restricted to the nuclei of Npr-2-positive cells due to a nuclear localization signal. In whole mounts and sagittal sections of heterozygous *Npr2*^*LacZ*/+^ mice Npr2-positive cells were found scattered throughout the complete mesencephalon in dorsal layers. In comparison to cGKI Npr2-positive cells were also found in the rostral mesencephalon and caudal prosomere 1 (Figures 1B_1_,_2_,C_2_). The rostral prosencephalon and the rostral prosomere 1 and prosomere 2 were negative at these stages. Additional expression of Npr2 was found at the border between mesencephalon and hindbrain (the isthmus) (Figures C_1_,_2_) and in the olfactory placode (Figure 1B_1_). Western blotting of subcellular fractions further confirmed expression of Npr2 and cGKI in brain regions as described above (Figure 1E).

**Figure 2:**
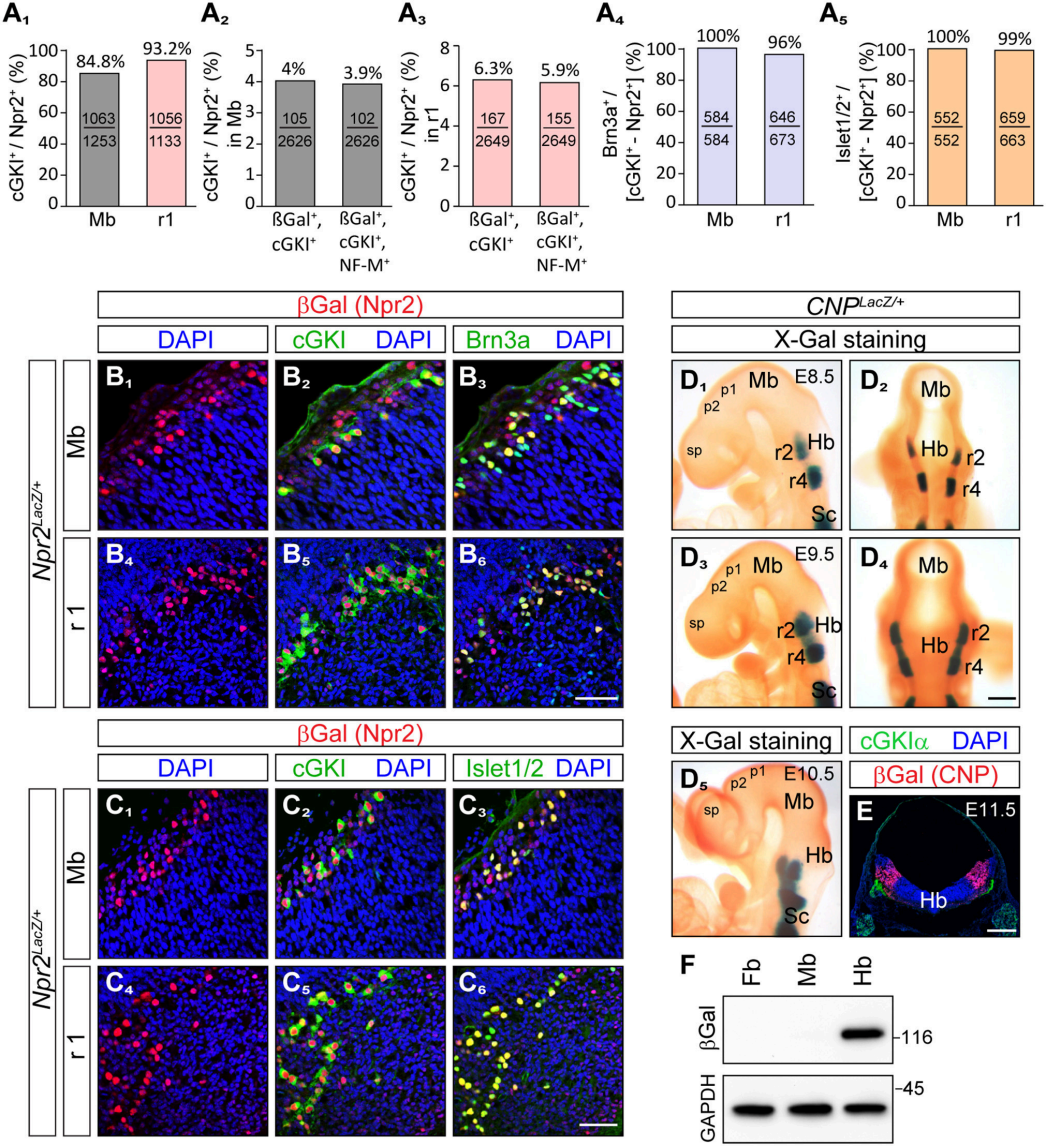
Brn3a and Islet1/2 co-localize with Npr2/cGKI in midbrain and rhombomere 1 and the ligand CNP is localized in the hindbrain but absent from the midbrain and forebrain. **(A**_1_**)** Percentage of cGKI-positive cells of Npr2-expressing cells in the mesencephalon and in rhombomere 1 at E12.5. **(A**_2_,_3_**)** Percentage of Npr2(β-Gal)/cGKI-positive or Npr2(β-Gal)/cGKI/neurofilament-positive cells of total midbrain or rhombomere 1 cells. Total cells were identified by DAPI. Anti-β-galactosidase staining was performed using the *Npr2-LacZ* reporter mouse line to monitor Npr2-expressing cells. **(A**_4_**,B)** Co-localization of Brn3a with Npr2(β-Gal)/cGKI-positive cells in the midbrain or rhombomere 1 at E12.5. **(A**_5_**,C)** Co-localization of Islet1 with Npr2(β-Gal)/cGKI-positive cells in the midbrain or rhombomere 1 at E12.5. The *Npr2-LacZ* reporter line was used to monitor expression of Npr2 in **(B, C)** by using an antibody to β-galactosidase. Scale bar for **(B**_1_**-C**_6_**)**, 50 μm. **(D)** Localization of CNP at E8.5, lateral **(D**_1_**)** and dorsal **(D**_2_**)** view of whole mounts. Expression analysis was done with the *CNP-LacZ* reporter mouse line using X-Gal staining. Localization of CNP at E9.5, lateral **(D**_3_**)** and dorsal **(D**_4_**)** view. **(D**_5_**)** Localization of CNP at E10.5 lateral view. Scale bar for **(D**_1_–**D**_5_**)** 30 μm. **(E)** Cross section of the hindbrain at the level of rhombomere 2 at E11.5 indicating that CNP is localized throughout all layers of the hindbrain. Anti-β-galactosidase staining of the *CNP-lacZ* reporter mouse line was used to monitor CNP. Scale bar, 250 μm. Hb, hindbrain; Mb, midbrain; Pros, prosencephalon; r2, rhombomere 2; r4, rhombomere 4; Sc, spinal cord. **(F)** Western blot of extracts of hindbrain, midbrain, and forebrain from *CNP-LacZ* reporter mice at E13.5 using anti-β-galactosidase to monitor expression of CNP. Equal loading was demonstrated by anti-GAPDH. Molecular mass markers are indicated at the right of the panel in kD.

Importantly, co-expression of Npr2 and cGKI - one characteristic feature of Npr2-mediated axon branching—was found in cells of the outer most layers of the caudal mesencephalon (Figures 1D_1_,_2_,_3_, 2B_2_,C_2_). Here, 84.8% of Npr2-positive cells express cGKI (Figure 2A_1_). An additional population co-expressing Npr2 and cGKI was detected in rhombomere 1 of the hindbrain (Figures 1D_2_,_4_, 2B_5_,C_5_) where 93.2% of Npr2-positive cells express cGKI (Figure 2A_1_). Neurons expressing both Npr2 and cGKI and in addition neurofilament represent 3.9 and 5.9% of total cells (DAPI-positive) in the midbrain and rhombomere 1 of the hindbrain, respectively (Figures 2A_2_,_3_).

The localization of their somata in the dorsal mesencephalon as well as the trajectory of their axons suggested that Npr2/cGKI-positive cells in the embryonic midbrain might represent MTNs. Indeed, a set of marker proteins that define MTNs including the LIM homeodomain protein Islet1 (Hunter et al., 2001; Ichikawa et al., 2005), the intermediate filament protein peripherin (Barclay et al., 2007) and the Pou homeodomain protein Brn3a (Fedtsova and Turner, 1995; Hunter et al., 2001) were found to completely co-localize with Npr2/cGKI-expressing neurons (Figures 2A_4_,_5_,B_1_-C_6_, 3A_1_).

**Figure 3:**
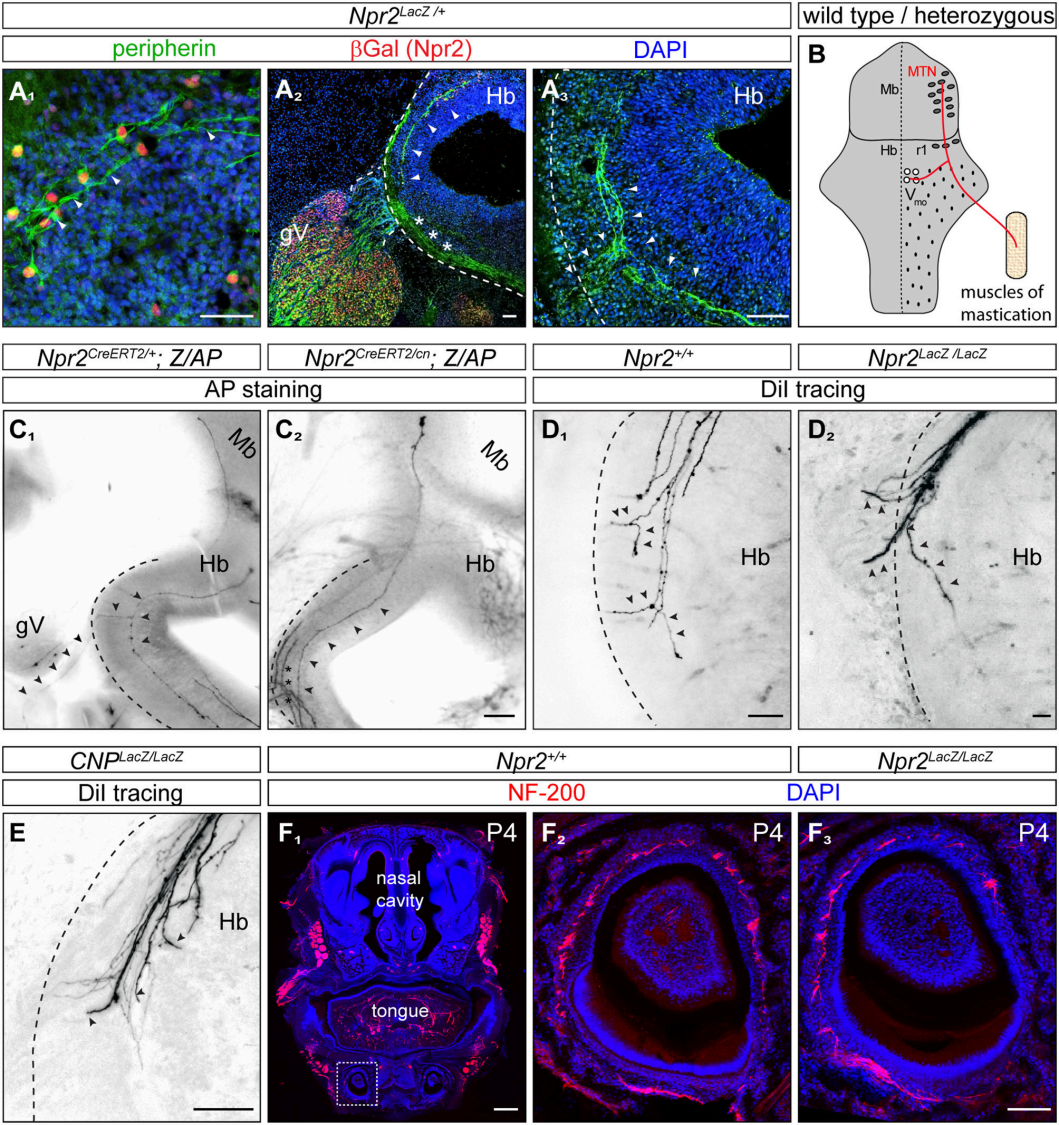
In the absence of Npr2 or CNP MTN axons fail to form Y-shaped bifurcations in rhombomere 2 of the hindbrain. **(A**_1_**)** Peripherin co-localizes with Npr2-positive cells in the midbrain and **(A**_2_,_3_**)** is found in descending MTN axons which extend in lateral regions of the hindbrain (arrow heads). Trigeminal axons are also positive for peripherin which grow at the lateral border of the hindbrain (asterisks). Anti-β-galactosidase staining monitors Npr2 expression in *Npr2-LacZ* reporter mouse. Scale bar 50 μm. **(B)** Scheme of bifurcating MTN afferents. Hb, hindbrain; Mb, midbrain; MTN, mesencephalic trigeminal neuron; r1, rhombomere 1; Vmo, trigeminal motor nucleus. **(C**_1_**)** An Npr2-positive axon originating in rhombomere 1 which bifurcates in rhombomere 2 from a heterozygous *Npr2* mutant. One arm is leaving the hindbrain via the root of the trigeminus demarcated by arrowheads growing toward V3 of the trigeminus (mandibular region). The other branch stays within the hindbrain to grow to Vmo. Another afferent originating in the midbrain had not reached the bifurcation zone (transgenic sparse labeling by alkaline phosphatase staining using the *Z/AP* reporter mouse line method). **(C**_2_**)** A MTN axon (arrow heads) bypassing the bifurcation zone without generating a peripheral branch from an *Npr2* knockout mutant (sparse labeling by using Z/AP). Trigeminal axons which are entering the hindbrain and which also do not bifurcate in global *Npr2* knockouts are marked by stars. Scale bar for **(C**_1, 2_**)** 200 μm. **(D**_1_**)** DiI tracing of MTN axons in the wild type hindbrain at the level of rhombomere 2. Two bifurcating axons are indicated by arrowheads. In 5 control embryos (*Npr2*^+/+^*, CNP*^+/+^ and *Npr2*^*CreERT*2/+^*;Z/AP*) 15 bifurcating MTN afferents were observed. No non-bifurcating afferents were found (see also Table 1). **(D**_2_**)** In the absence of Npr2 MTN afferents do not bifurcate and either exit or extend further in the hindbrain. Single non-bifurcating axons and fascicles of axons are shown. Scale bars in **(D**_1_,_2_**)** 50 μm. **(E)** In the absence of the ligand CNP no bifurcating MTN axons were observed at the level of the dorsal root of the trigeminus. **(D**_2_**,E)** In 4 homozygous mutants (*CNP*^*Lacz*/*LacZ*^*, Npr2*^*LacZ*/*LacZ*^, and *Npr2*^*CreERT2*/*cn*^;*Z/AP*) 40 non-bifurcating and 4 bifurcating MTN afferents were observed (see also Table 1). Scale bar, 50 μm. **(F)** Neurofilament staining of a transversal section of a tooth at P4 show less axons in the absence of Npr2. **(F**_1_**)** Overview of a transversal section of the mouth at P4; neurofilament staining of wild type **(F**_2_**)** and Npr2 knockout **(F**_3_**)**. Scale bar in **(F**_1_**)**, 500 μm; in **(F**_2, 3_**)**, 100 μm. The square in **(F**_1_**)** denotes the region enlarged in **(F**_2_**)**.

Thus, we conclude that Npr2/cGKI-positive cells in the caudal mesencephalon represent MTNs. The cGKI/Npr2-positive neurons in rhombomere 1 contribute to the population of MTNs in the fully differentiated brain and might arise by migration from the mesencephalon as has been previously proposed (Widmer et al., 1998; Lazarov, 2002; Espana and Clotman, 2012).

### The Npr2 ligand CNP is present in rhombomere 2 where MTN afferents bifurcate

Expression of the ligand CNP that binds and activates the receptor guanylyl cyclase Npr2 was studied using a *CNP-LacZ* reporter mouse that has a *LacZ* expression cassette with a nuclear localization signal knocked-in into the *CNP* gene locus. In contrast to cGKI and Npr2, CNP was absent from mesencephalon and also from forebrain and rhombomere1 but was expressed in rhombomere 2 and 4 of the hindbrain already at E8.5 and at E9.5 (Figures 2D,E). Beginning at E9.5, CNP also became expressed in rhombomere 3 and from E10.5 on it could be detected in more caudal parts of the hindbrain. Cross-sections of the hindbrain at the level of rhombomere 2 indicated that all cell layers of the hindbrain—from the outer margin to the ventricle—are expressing CNP (Figure 2E). Western blotting of extracts from hindbrain, mesencephalon or forebrain further confirmed expression of CNP-LacZ in the hindbrain and not in the midbrain or forebrain at these embryonic stages (Figure 2F). Therefore, descending Npr2-positive MTN afferents initially extend through a CNP-free territory but then might encounter CNP in rhombomere 2. Here, activation of Npr2 might result in the splitting of their axon in two arms as reported for DRG and CSG axons.

### Afferents of MTN fail to split into a central and peripheral branch in CNP- or Npr2-deficient mice

Previously published intra-axonal injections of horseradish peroxidase in axons of adult cats, rats or snakes revealed that MTN axons split in a peripheral and central branch in the hindbrain (Dacey, 1982; Shigenaga et al., 1988; Dessem and Taylor, 1989; Tsuru et al., 1989; Luo et al., 1991). The peripheral afferent exits the hindbrain through the trigeminal root to grow to the jaw while the central branch travels caudally to the trigeminal motor nucleus (Vmo) (Lazarov, 2007) (see scheme in Figure 3B). To analyze whether CNP or Npr2 are involved in this Y-shaped bifurcation of MTN axons the path of individual MTN afferents in the hindbrain was traced. We applied two independent axon tracing methods: (1) DiI axon labeling (Honig and Hume, 1989) and (2) a transgenic method for sparse labeling of Npr2-positive neurons (Schmidt et al., 2013). In the hindbrain MTN afferents (marked by arrowheads in Figures 3A_1_–A_3_) form a lateral tract but do not intermingle with ingrowing and bifurcating axons of the trigeminal ganglion (marked by asterisks in Figure 3A_2_) as detected by anti-peripherin staining (Figures 3A_2_,_3_). Individual axons were analyzed in vibratome sections of the hindbrain and in the case of DiI tracings in z-stacks of confocal images. In control embryos *(Npr2*^*CreERT2*/+^*;Z/AP or Npr2*^+/+^*)* MTN afferents bifurcate at the level of the trigeminal ganglion (Figures 3C_1_,D_1_; see also Video S1). In the absence of Npr2 (*Npr2*^*CreERT2*/*cn*^*;Z/AP, Npr2*^*LacZ*/*LacZ*^) or CNP *(CNP*^*LacZ*/*LacZ*^*)* MTN axons were not able to form Y-like branches in rhombomere 2 (Figures 3C_2_,D_2_,E and Table 1; see also Video S2). In homozygous *Npr2* or *CNP* mutants MTN afferents either turned into the direction of the trigeminal root or extend caudally within the hindbrain. Consequently, less neurofilament-positive structures surrounding teeth of the mandible were detected (Figure 3F). In conclusion, MTN afferents use Npr2-mediated cGMP signaling to split into a peripheral and a central branch similarly as afferents from DRG or CSG when entering the spinal cord or hindbrain, respectively.

**Table 1:** Impaired MTN afferent bifurcation in the absence of Npr2 or CNP.

**CONTROLS AND GLOBAL KNOCKOUTS**							
***CNP**^+/+^*		***CNP**^*LacZ*/*LacZ*^*		***Npr2**^+/+^* **and** ***Npr2**^*CreERT*2/+^**;Z/AP***		***Npr2**^*LacZ*/*LacZ*^* **and** ***Npr2**^*CreERT*2/*cn*^**;Z/AP***	
Control		Knockout		Control		Knockout	
**Bifur**	**Turn**	**Bifur**	**Turn**	**Bifur**	**Turn**	**Bifur**	**Turn**
7 (3)	0 (3)	3 (2)	26 (2)	8 (2)	0 (2)	1 (2)	14 (2)
**CONTROL AND CONDITIONAL KNOCKOUTS**							
***Npr2**^*flox*/*flox*^*		***Npr2**^*flox*/*flox*^**; Wnt1**^*Cre*^*		***Npr2**^*flox*/*flox*^**; Engr1**^*Cre*^*	
Control		Co.knockout		Co.knockout	
**Bifur**	**Turn**	**Bifur**	**Turn**	**Bifur**	**Turn**
2 (3)	0 (3)	0 (3)	14 (3)	1 (5)	27 (5)

*Total numbers of individual MTN axons that could be identified by DiI tracing or sparse labeling using the Npr2^CreERT2^;Z/AP transgene. The first number indicates the number of individual axons identified and the second number in parenthesis the numbers of embryos analyzed. In control animals (CNP^+/+^, Npr2^+/+^, Npr2^CreERT2/+^;Z/AP and Npr2^flox/flox^) 17 bifurcating and 0 non-bifurcating (“turn”) axons were counted in 8 embryos. In homozygous mutants (CNP^LacZ/LacZ^, Npr2^LacZ/LacZ^, Npr2^CreERT2/cn^;Z/AP, Npr2^flox/flox^;Wnt1^Cre^ and Npr2^flox/flox^;Engr1^Cre^) 5 bifurcating and 81 non-bifurcating axons were counted in 12 embryos*.

### Maximal biting force is reduced in the absence of MTN axon bifurcation

**Figure 4:**
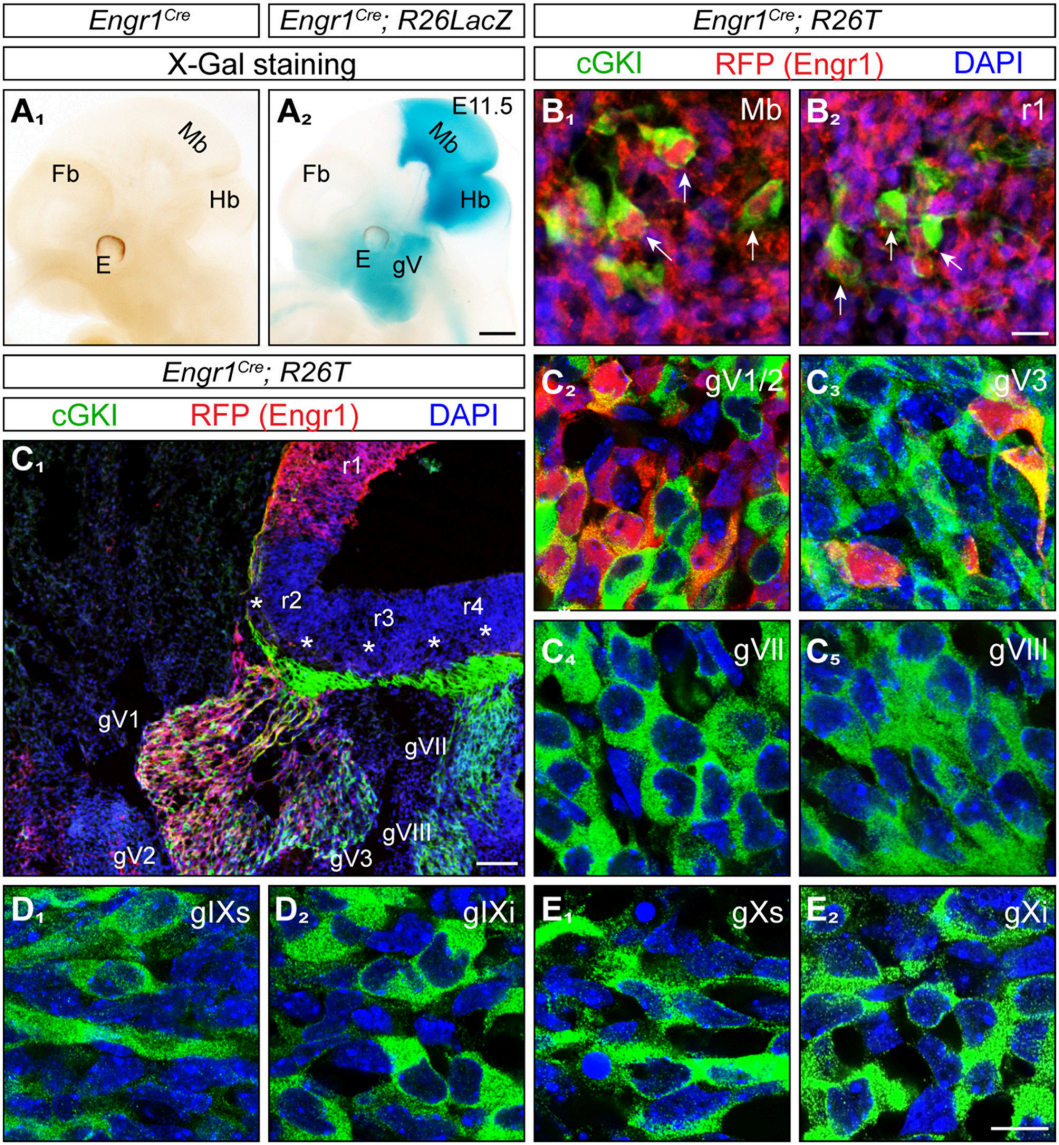
Expression of Engr1-Cre in the midbrain, hindbrain and trigeminal ganglion. **(A**_1_,_2_**)** Detection of Engr1-Cre expression in whole mounts at E11.5 using X-Gal staining of the conditional *R26LacZ* reporter activated by Engr1-Cre. Strong expression is observed in the midbrain, isthmus and rhombomere 1 and to a lesser extent in the trigeminal ganglion and in the region surrounding the mouth. Scale bar, 500 μm. **(B**_1_,_2_**)** Expression of Engr1-Cre in cGKI-positive cells (MTNs, indicated by arrows) in the mesencephalon or in rhombomere 1, respectively, at E11.5. Engr1-Cre is detected in red by using the conditional reporter line *R26T* which is activated by Engr1-Cre and counterstained by antibodies to the red fluorescent protein, cGKI in green and DAPI in blue. Scale bar, 10 μm. **(C)** Expression of Engr1-Cre in gV (overview in **C**_1_) and at the cellular level in gV1/2 (V1 - ophthalmic and V2 maxillary part) **(C**_2_**)** and gV3 (mandibular part) **(C**_3_**)** at 11.5. Asterisks in **(C**_1_**)** depict entry zones of CSGs in the hindbrain. r, rhombomere. Engr1-Cre is detected in red, cGKI in green and DAPI in blue. Scale bar in **(C**_1_**)**, 100 μm. **(C**_4_**-E**_2_**)** No expression of Engr1-Cre 1 in gVII, gVIII, gIX, und gX. Engr1-Cre is detected in red, cGKI in green and DAPI in blue. Scale bar in **(C**_2_**-E**_2_**)** 20 μm.

Next, we asked whether the lack of axon bifurcation of MTN afferents has functional consequences at adult stages. Due to technical difficulties in recording jaw movements for coordination in mice (Koga et al., 2001) we analyzed the maximal bite force in a series of bites of *Npr2* mutants to characterize physiological consequences of the absence of axon bifurcation of MTN. However, the dwarfed appearance of constitutive Npr2-deficient mice (Chusho et al., 2001; Tamura et al., 2004; Tsuji and Kunieda, 2005; Ter-Avetisyan et al., 2014) and their decreased survival at post weaning stages, prevents the use of *Npr2* global mouse knockouts for measurement of bite force. Therefore, a strategy for conditional inactivation of Npr2 in brain regions by cross-breeding of the *Npr2-flox* mouse line (Tröster et al., 2018) with a Cre-driver was developed. The transcription factor engrailed1 has been previously shown to be selectively expressed in the mesencephalon and rhombomere 1 at very early embryonic stages (Davis and Joyner, 1988; Sapir et al., 2004; Basson et al., 2008) and therefore *Engr1-Cre* might be a suitable candidate to remove Npr2 from MTN without inducing additional phenotypes observed in the global *Npr2* knockout. To further review the localization of Engr1-Cre the *Engr1-Cre* encoding mouse line (Kimmel et al., 2000) was crossed with the *R26-LSL-tdTomato* (R26T) or the *R26-LacZ* reporter mouse lines to monitor the localization of Engr1-Cre in whole mounts and at the single cell level. As expected, we observed a strong overall pattern of Engr1-Cre expression in the midbrain and rhombomere 1 (Figures 4A_1_,_2_) including cGKI-positive MTN (Figures 4B_1_,_2_).

Unanticipated, we also detected Engr1-Cre in the trigeminal ganglion (gV) in addition to the localization in the mesencephalon and rhombomere 1 (Figure 4A_2_). The trigeminal ganglion also expresses Npr2 (Ter-Avetisyan et al., 2014) and its central afferents bifurcate by Npr2-mediated signaling in rhombomere 2. Moreover, neurons of the trigeminus also functionally overlap to some degree with MTN in conveying sensory information from facial regions (Baker and Bronner-Fraser, 2001; Erzurumlu et al., 2010). Analysis at the cellular level in sections detected expression of Engr1-Cre primarily in neurons of the trigeminus that generate axons of the maxillary (V2) and ophthalmic (V1) nerve whereas only a small population of mandibular (V3) neurons expresses Engr1-Cre (Figures 4C_1_–C_3_). Other CSGs (gVII/VIII, gIX, and gX) that are known to express Npr2 and cGKI were negative for Engr1-Cre (Figures 4C_4_-E_2_). Overall, the localization indicated that Engr1-Cre is a suitable candidate to conditionally inactivate Npr2 in cells of the mesencephalon and rhombomere 1.

**Figure 5:**
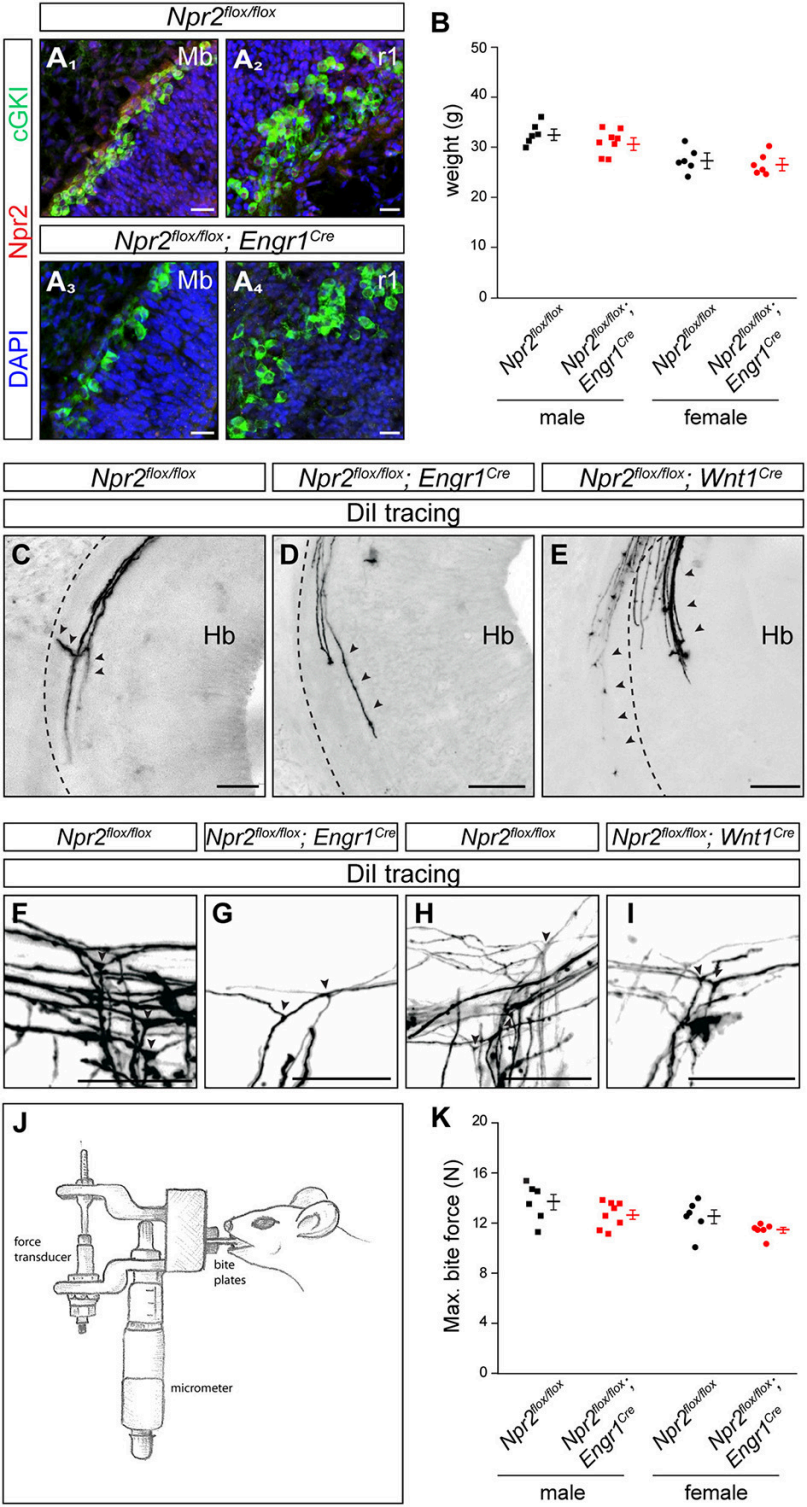
In the absence of MTN axon bifurcation a decreased maximal bite force was measured in *Npr2*^*flox*/*flox*^*;Engr1*^*Cre*^ in comparison to control mice. **(A)** Strong reduction of Npr2 was observed in sagittal sections of midbrain or rhombomere 1 in conditional *Npr2*^*flox*/*flox*^*;Engr1*^*Cre*^ mutants at E12.5. Scale bar, 20 μm. **(B)** Body weight of 15 weeks old *Npr2*^*flox*/*flox*^ control and *Npr2*^*flox*/*flox*^*;Engr1*^*Cre*^ mutant mice (scatter blots). Body mass differed between males and females [*F*_(1, 22)_ = 24.84; *P* < 0.001]. However, body mass did not differ between the control and experimental groups [*F*_(1, 22)_ = 2.55; *P* = 0.13]. The interaction effect was also not significant [*F*_(1, 22)_ = 0.60; *P* = 0.45]. **(C)** A bifurcating MTN axon in rhombomere 2 in the *Npr2*^*flox*/*flox*^ line (control). Arrowheads point to a bifurcating MTN afferent. **(D,E)** MTN afferents do not bifurcate in the hindbrain in rhombomere 2 in conditional *Npr2* mutants (*Npr2*^*flox*/*flox*^*;Engr1*^*Cre*^ or *Npr2*^*flox*/*flox*^*;Wnt1*^*Cre*^). The broken lines indicate the margin of the hindbrain and arrowheads point to MTN afferents. In homozygous mutants (*Npr2*^*flox*/*flox*^*;Engr1*^*Cre*^ and *Npr2*^*flox*/*flox*^*;Wnt1*^*Cre*^) 41 non-bifurcating and 1 bifurcating MTN afferent were observed in 8 embryos (see also Table 1). Scale bar for **(C–E)**, 50 μm. **(F–I)** DiI tracings of trigeminal afferents in *Npr2*^*flox*/*flox*^*;Engr1*^*Cre*^ or *Npr2*^*flox*/*flox*^*;Wnt1*^*Cre*^ show normal bifurcation in the hindbrain at E12.5. In 7 control embryos (*Npr2*^*flox*/*flox*^) 29 bifurcating trigeminal afferents and in 7 mutant embryos (*Npr2*^*flox*/*flox*^*;Engr1*^*Cre*^ and *Npr2*^*flox*/*flox*^*;Wnt1*^*Cre*^) 37 bifurcating and 3 non-bifurcating trigeminal afferents were observed (see also Table 2). Scale bar, 50 μm. **(J)** Schematic drawing of the bite force measurement instrument. **(K)** Scatter blots of the maximal bite force in Newton (N) of *Npr2*^*flox*/*flox*^ (control) and Npr2^*flox*/*flox*^*;Engr1*^*Cre*^ mutant mice (15 weeks old). The maximal bite force of males was greater than in females [*F*_(1, 22)_ = 6.61; *P* = 0.017]. Bite force also differed between mutants and control groups [*F*_(1, 22)_ = 4.65; *P* = 0.042] with bite force being reduced in conditional *Npr2* mutants. Interaction effects were not significant, however [*F*_(1, 22)_ = 0.02; *P* = 0.88]. *Npr2*^*flox*/*flox*^, *n* = 6 male and *n* = 6 female; *Npr2*^*flox*/*flox*^*;Engr1*^*Cre*^, *n* = 8 male and *n* = 6 female.

Inactivation of Npr2 in MTNs by Engr1-Cre-mediated excision of exon 17 and 18 of the *Npr2* gene resulted in strongly reduced expression of Npr2 protein in MTNs as demonstrated by antibodies to the extracellular domain of Npr2 (compare Figure 5A_1_ with Figure 5A_3_ for midbrain and Figure 5A_2_ with Figure 5A_4_ for rhombomere 1). Conditional *Npr2* mutants (*Npr2*^*flox*/*flox*^*;Engr1*^*Cre*^) developed normally as demonstrated by their body weight (Figure 5B). Body mass differed between sexes [*F*_(1, 22)_ = 24.84; *P* < 0.001] with males being heavier than females. However, body mass did not differ between control and experimental groups [*F*_(1, 22)_ = 2.55; *P* = 0.13]. The interaction effect between sex and experimental group was also not significant [*F*_(1, 22)_ = 0.60; *P* = 0.45]. No decrease in the survival rate or any problems on the health status of 21 inspected *Npr2*^*flox*/*flox*^*;Engr1*^*Cre*^ animals in which Npr2 was conditionally inactivated was observed.

DiI axon tracing at embryonic day 12.5 revealed bifurcation errors of MTN axons in *Npr2*^*flox*/*flox*^*;Engr1*^*Cre*^ as described for the global mouse knockouts of *CNP* or *Npr2*. Only extensions within the hindbrain or into the direction of the trigeminal ganglion were detected (Figures 5C–E and Table 1). Since neurons of the trigeminus are also implicated in orofacial sensation, bifurcation of their afferents was inspected in the hindbrain. In contrast to global *Npr2* knockout mice (Ter-Avetisyan et al., 2014) bifurcation of trigeminal afferents entering the hindbrain in *Npr2*^*flox*/*flox*^*;Engr1*^*Cre*^ is normal (Figures 5F,G and Table 2). Similar results were obtained when Npr2 was conditionally inactivated with *Wnt1-Cre* which is also expressed in the midbrain but also in all neural crest derived sensory neurons at early stages (Echelard et al., 1994; Danielian et al., 1998). Again, branching errors were observed for MTN afferents (Figure 5E and Table 1) but not for trigeminal central afferents (Figures 5H,I and Table 2).

**Table 2:** Bifurcation of afferents from the trigeminal ganglion (gV) in the absence of Npr2 in conditional mutants is normal (DiI axon tracing).

***Npr2***^***flox/flox***^		***Npr2***^***flox/flox***^***;Wnt1***^***Cre***^		***Npr2***^***flox/flox***^***;Engr1***^***Cre***^	
Control		Co.knockout		Co.knockout	
**Bifur**	**Turn**	**Bifur**	**Turn**	**Bifur**	**Turn**
29 (7)	0 (7)	9 (2)	1 (2)	28 (5)	2 (5)

Bite force measurements were done using a piezoelectric force transducer attached to a handheld charge amplifier (see scheme in Figure 5J). Biting on incisors was recorded in two independent sessions including four bites each (Aguirre et al., 2002). From these experiments the single highest bite force was statistically analyzed. Our analysis indicated that the maximal bite force differed between sexes with males biting harder than females [*F*_(1, 22)_ = 6.61; *P* = 0.017]. Bite force also differed between experimental (*Npr2*^*flox*/*flox*^*;Engr1*^*Cre*^) and control groups (*Npr2*^*flox*/*flox*^) [*F*_(1, 22)_ = 4.65; *P* = 0.042] with bite force being significantly reduced in conditional *Npr2* mutants (Figure 5K). Interaction effects between sex and experimental groups were not significant, however [*F*_(1, 22)_ = 0.02; *P* = 0.88]. Malocclusion of the upper and lower incisors detected in some *Npr2*^*flox*/*flox*^*;Wnt1*^*Cre*^ mutants (Tröster et al., 2018) prevented them from being tested in biting experiments.

In conclusion, our data indicated that the maximal biting force was significantly reduced in conditional *Npr2* mutants most likely due to an impaired bifurcation of MTN afferents. A role of the trigeminus on these biting measurements can be excluded since bifurcation of trigeminal afferents was not affected in *Npr2*^*flox*/*flox*^*;Engr1*^*Cre*^ mice. The natural difference between males and females in their maximal bite force was retained in mutants and controls (Thomas et al., 2015). Overall, our data suggest that sensory feedback to control bite force might be impaired by the absence of MTN afferent bifurcation.

## Discussion

**Figure 6:**
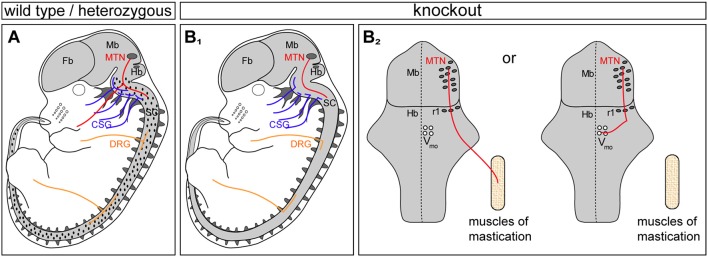
Three types of sensory neurons form T- or Y-shaped branches controlled by CNP/Npr2-mediated cGMP signaling: MTN, CSG, and DRG. **(A)** Scheme summarizing previous data and this study on the expression profile of Npr2/cGKI in embryonic neurons: MTN, CSG, and DRG are positive for both components. Localization of the ligand CNP in the hindbrain and dorsal spinal cord is illustrated in dots in **(A)**. **(B**_1_**)** Absence of the CNP/Npr2 signaling system caused axon bifurcation errors in these three types of neurons. CSG, cranial sensory ganglia; DRG, dorsal root ganglia; Fb, forebrain; Mb, midbrain; MTN, mesencephalic trigeminal neurons; Hb, hindbrain; r1, rhombomere1; SC, spinal cord; Vmo, trigeminal motor nucleus. **(B**_2_**)** Scheme of the circuit of major axon branches of MTN in *Npr2* mutants illustrating the reduced sensory feedback to motor neurons in Vmo of the hindbrain. See also Figure 3B for wild type.

In this report we identified MTNs as an additional neuronal population that similar to DRG- or CSG-neurons co-express Npr2 and cGKI at early developing stages when their afferents are projecting into the hindbrain (A scheme shows the location of CNP, Npr2, and cGKI in Figure 6A and the bifurcation errors are illustrated in Figure 6B_1_). The ligand CNP which binds and activates the receptor guanylyl cyclase Npr2 is not found in the mesencephalon but is expressed in the hindbrain in rhombomere 2. The timing and pattern of expression of CNP in the hindbrain is in line with the arrival of MTN afferents to split into two major branches. Of note is that this region overlaps with the entrance zone of trigeminal axons that also bifurcate by CNP/Npr2-mediated signaling although more laterally when entering the hindbrain. In the absence of Npr2-mediated cGMP-signaling—in global as well in conditional mouse mutants—the formation of Y-shaped afferent branches of MTN is lacking. Consequently, MTN afferents either exit the hindbrain via the root of the trigeminal ganglion or grow further caudally within the hindbrain. The absence of bifurcation results in two possible scenarios for the growth of the stem afferent: MTN afferents project either to motor neurons in the hindbrain or extend to the mandible (A scheme in Figure 6B_2_ illustrates these two options). Under normal conditions afferent impulses from the sensory ending propagate along the peripheral afferent to the Y-shaped bifurcation junction and thereafter propagate along the two branches separately to the MTN soma and to the presynaptic terminal on the motor neuron. Therefore, in the absence of bifurcation sensory information conducted by an individual MTN might not directly reach the motor neuron pool.

MTNs process sensory information from the spindles of the masseter and temporalis muscles or the periodontal mechanoreceptors around teeth and project to the trigeminal motor nucleus and related nuclei. These motor neurons are the output neurons of the brainstem responsible for a variety of orofacial motor functions. The main role of MTN is to provide sensory feedback from the jaw closing muscles to trigeminal motor neurons in the brainstem. Sensory feedback is essential to adjust these movements by providing information about food hardness and elasticity and contraction properties of the jaw closing muscles. Reduction of sensory feedback from the jaw might limit the quality by which commands of motor neurons from Vmo can be refined.

Since there are technical difficulties in recording jaw movements during mastication in small animals such as mice (Koga et al., 2001) we restricted our analysis to the measurement of the maximal bite force to begin to understand the physiological consequences of the absence of axon bifurcation in MTN afferents. Using Engrailed1-Cre we observed a significantly reduced maximal biting force in the absence of MTN axon bifurcation in female as well as in male conditional mutants of Npr2. In this context it is important to note that the bifurcation of trigeminal axons is not perturbed in Npr2 mutants conditionally inactivated by Engr1-Cre. Therefore, the reduced maximal biting force might primarily result from bifurcation errors in the MTN system. On the one hand, our observations might not be unexpected since neuronal regulation of mastication and biting might continuously require sensory feedback from the innervated masticatory muscles and teeth to enable real-time adjustment of movements of jaw muscles and to counteract unexpected perturbations (Yamada et al., 2005; Westberg and Kolta, 2011). This fast sensory feedback appears to be realized by the MTN circuit which establishes a link to motor neurons without further intercalated interneurons. The most likely interpretation of the decreased masticatory force might be the reduced information on muscle stretch reaching motor neurons is due to deficits in the innervation of motor neurons as the main effector of biting strength (Figure 6B_2_). On the other hand the reduction in biting force is remarkable since proprioceptive responses for motor coordination are not affected when axon bifurcation is abolished in DRG neurons of *Npr2*^*flox*/*flox*^*;Wnt1*^*Cre*^ mutants (Tröster et al., 2018). In the spinal cord proprioceptive circuits of muscle spindles might contain intercalated interneurons that communicate with spinal motor neurons in contrast to the MTN-motor neuron circuit (Azim et al., 2014).

DRG neurons, CSG neurons and MTNs are pseudo-unipolar primary afferent neurons, however, MTN are unique with respect to the localization of their cell bodies within the CNS. Whereas DRG and CSG are generated from neural crest cells or from transient focal thickenings of the ectoderm in the head region, MTN emerge from the dorsal mesencephalon and not from neural crest cells that invade the mesencephalon as previously thought (Hunter et al., 2001; Dyer et al., 2014; Lipovsek et al., 2017). In spite of their location in the CNS and their different origin, MTNs maintain some characteristics of other sensory neurons including the expression of Npr2 and cGKI and bifurcation of their afferent. MTN also receive abundant synaptic inputs on their somata from other parts of the brain including the hypothalamus, amygdala, raphe nuclei, locus coeruleus, habenula and the pontine reticular formation (Lazarov, 2007; Ohara et al., 2016). These observations suggest that the sensory activity of MTN can be modulated by multiple brain regions and indicates that MTNs serve not only as primary sensory neurons but also as interneurons in the regulation of mastication. MTN are crucial for controlling the strength of occlusion and the position of the mandible.

CNP and Npr2 are also involved in the process of endochondral ossification which is essential for long bone growth. Therefore, in humans and in mice loss-of-function mutations in the *NPR2* gene cause a skeletal dysplasia, termed acromesomelic dysplasia type Maroteaux (AMDM; OMIM602875) with an extremely short and disproportionate stature (Bartels et al., 2004; Potter, 2011; Kuhn, 2016). AMDM is a rare autosomal-recessive genetic disorder with a prevalence of one out of 1,000,000 individuals. In contrast, *NPR2* gain-of-function mutations result in an overgrowth syndrome (Miura et al., 2014). Currently, it can only be hypothesized whether the absence of the Npr2-mediated cGMP signaling in MTNs in these patients causes branching errors of MTN axons within the hindbrain and it has not been investigated whether AMDM patients have defects in mastication or swallowing. Our data using mouse genetics indicated deficits in the biting strength in the absence of Npr2. These observations might provide a framework for future studies to characterize deficits in jaw-closing muscles of human patients with mutations in the *NPR2* gene and might help to analyze possible dysphagia. Such studies might reveal whether the absence of bifurcation not only controls the strength of occlusion but also the position of the mandible - experiments which are difficult to perform with mice.

## Author contributions

GT-A, AD, AH, HS, JS, SA, and FR performed experiments and evaluated data. GT-A, AD, HS, and FR contributed to the writing of the manuscript.

### Conflict of interest statement

The authors declare that the research was conducted in the absence of any commercial or financial relationships that could be construed as a potential conflict of interest.

## Supplementary material

Video S1 A series of z-stacks of confocal images of DiI tracings of wild type MTN afferents at the level of rhombomere 2 are compiled. The interval is 2 μm.

Video S2 A series of z-stacks of confocal images of DiI tracings of *Npr2* knockout MTN afferents at the level of rhombomere 2 are compiled. The interval is 10.7 μm.
